# Acceptance and willingness to pay for COVID-19 vaccine among school teachers in Gondar City, Northwest Ethiopia

**DOI:** 10.1186/s41182-021-00337-9

**Published:** 2021-08-09

**Authors:** Kegnie Shitu, Maereg Wolde, Simegnew Handebo, Ayenew Kassie

**Affiliations:** Department of Health Education and Behavioral Sciences, Institute of Public Health, College of Medicine and Health Sciences, University of Gondar, Gondar, Ethiopia

**Keywords:** Willingness to pay, COVID-19, Ethiopia

## Abstract

**Background:**

Vaccination is one of the strategies to prevent the COVID-19 pandemic. COVID-19 vaccine acceptance and cost are important factors affecting its uptake. However, little is known about acceptance and willingness to pay (WTP) for the vaccine.

**Objective:**

This study aimed to assess acceptance and WTP for the COVID-19 vaccine and its associated factors among school teachers.

**Methods:**

An institution-based cross-sectional study was conducted among school teachers. A stratified simple random sampling technique was employed to select the participants. The contingent valuation method was used to assess each respondent’s willingness to pay for the COVID-19 vaccination. A multivariable logistic regression analysis was employed to identify significant factors associated with WTP for the COVID-19 vaccine. A p value of less than 0.05 and a 95% confidence interval were used to declare statistical significance.

**Result:**

A total of 301 school teachers participated with a response rate of 99.6%. The mean age of participants was 39.5 (SD ± 8.7 years). The mean WTP for the COVID-19 vaccine is US$ 4.98 (± US$ 1.6). Less than half (40.8%) of participants were willing to accept the COVID-19 vaccine whereas only 36.9% (95% CI, (31.6%, 42.5%)) of them were WTP for the vaccine. Being male (AOR = 3.23; 95% CI, (1.70, 6.14)), being a private school teacher (AOR = 3.27; 95% CI, (1.76, 6.10)), having high perceived susceptibility to COVID-19 (AOR = 2.69; 95% CI, (1.38, 5.24)), having high perceived seriousness of COVID-19 (AOR = 4.04; 95% CI, (1.80, 9.1)), and having high perceived benefit of COVID-19 vaccine (AOR = 3.0; 95% CI, (1.41, 6.34) were significant factors associated with WTP for COVID-19 vaccination.

**Conclusion and recommendation:**

The magnitude of acceptance and WTP for the COVID-19 vaccine was low among school teachers. Male sex, private school affiliation, high perceived risk of COVID-19, and benefit of COVID-19 vaccine were the most important factors affecting school teacher’s WTP for COVID-19 vaccine. Thus, health communication programs should give due emphasis to raising teacher’s perceived risk of COVID-19 and the benefit of the COVID-19 vaccine to improve their acceptance and WTP for the vaccine. Furthermore, subsidizing the cost of the vaccine may improve their WTP.

## Introduction

COVID-19 is a virus that is very contagious with a fast-spreading rate to every part of the world. As of 30 March 2021, about 127,349,248 confirmed cases of COVID-19, including 2,787,593 deaths have been reported worldwide. Newly reported cases are increasing almost in all parts of the world since the last 2 weeks of March 2021 [1, 2].

Daily new COVID-19 infections reported in Ethiopia each day reaches a new high: As of 27 March 2021, about 2142 new cases and 15 new deaths bring the total cases and death to be 198,794 and 2784 respectively [3]. Regardless of this increment, preventive measures taken by the people are not satisfactory to tackle the pandemic [4, 5]. In this point of view, COVID-19 vaccination has a crucial role to reduce the transmission of the pandemic.

Various studies done in different parts of the world have been reported different levels of acceptance/willingness to pay (WTP) for COVID-19. For instance, a survey on willingness to vaccinate against COVID-19 in Australians revealed 4.9% said they would not get the vaccine whereas 85.8% of the participants are willing to get the vaccine if it became available [6].

There is a relatively high vaccine acceptance rate for COVID-19 vaccines in Chile, where a study claimed 90.6% of the general public would be willing to pay for a COVID-19 vaccine. In this study, only 55% of individuals are willing to pay the initial value and a second value, higher than the first; whereas 12% who said “yes” to the first value, are not willing to pay for the second higher value [7]. Another study from Indonesia revealed that 78.3% of participants were willing to pay for the COVID-19 vaccine with a mean and median willingness to pay of US$ 57.20 and US$ 30.94 respectively [8]. A similar study in Malaysia reported that the mean amount that participants were willing to pay for a dose of the COVID-19 vaccine was US$30.66 [9].

From preexisted literature, willingness to pay for vaccination against COVID-19 was found to be associated with various factors. For instance, the study done in Australia revealed that reluctance to be vaccinated against COVID-19 related to inadequate health literacy and lower education level were significant factors associated with COVID-19 vaccination [6]. Another study from Chile reported that willingness to pay depends on the preexistence of chronic disease, knowledge of COVID-19, being sick with COVID-19, perception of government performance, employment status, income, health care, adaptation to quarantine with children at home, and whether the person has recovered from COVID-19 [7]. Moreover, in Indonesia being a healthcare worker, having a high income, and having high perceived risk were associated with a higher willingness to pay [8].

Nowadays, vaccine hesitancy is becoming a common phenomenon because people are highly affected by untrusted messages transmitted via various channels (social media) [10]. In turn, this affects vaccine uptake and coverage directly. Given this, working intensively on messaging about the vaccine needs to be done well before the vaccine is available [11]. To do so, determining the level of acceptance and perceptions of the vaccine is somewhat vital to design a tailored vaccine communication program.

However, how developing countries should accept/pay for COVID-19 vaccine is not well investigated even if it is very important to know. High levels of vaccination coverages are a key indicator of vaccine acceptance. Therefore, building public confidence in vaccines is an important starting point toward acceptance and sufficient uptake of safe COVID-19 vaccines. In this regard, identifying and addressing possible gaps and COVID-19 vaccine-related misconceptions in the public is a key element to be taken into consideration [12]. Hence, this study aimed at assessing the acceptance and willingness to pay for COVID-19 and associated factors among school teachers in Gondar City. To the best of the authors’ knowledge, this study is not conducted in Ethiopia. Thus, the study also gives insights to the policymakers, vaccine developers, and distributors. Also, it may ignite a new insight for further studies that might be conducted on the study topic for example research on the strategies to leave this work.

## Methods and materials

### Study design and setting

A school-based cross-sectional study was conducted among primary and secondary school teachers in the Gondar City from December 2020 to February 2021. The city is located about 727 km from Addis Ababa, the capital of Ethiopia, and 180 km from Bahir Dar, the capital of Amhara State. At 2019, the total population of the city was estimated to be 500,788, of which 300,000 were men and 200,788 were women. In the administration of the city, 3000 teachers are working in seventeen elementary and secondary schools of the city.

### Population

The study participants were primary and secondary school teachers from both private and public schools in Gondar City. Teachers who were not in Gondar City throughout the data collection period; and those who were severely ill to the extent they were not able to fill the questionnaire were excluded from the study.

### Sample size determination and sampling method

The sample size was determined using a single population proportion formula, where the following assumptions were considered: P (78.3%, proportion of willingness to pay from the previous study in Indonesia [8]), d (margin of error = 5%), and Zα/2 (the value of the standard normal curve score corresponding to the given confidence interval = 1.96) corresponding to 95% confidence level. Indeed, finite population correction formula was employed because the study population was less than 10, 000 and a 5% non-response rate was considered. Given this, the final sample size was computed to be 302.

A stratified simple random sampling technique was used to recruit the study participant. Firstly, stratification was done based on ownership of the schools into private (five schools) and public (twelve schools). Then, four governmental and two private schools were selected using the lottery method and the sample was proportionally allocated to each stratum. Finally, study participants were selected randomly using computer-generated random numbers.

### Data collection procedure

Data were collected using a pretested structured self-administered questionnaire prepared by the investigators by reviewing different literature [13–18]. In the very beginning, the tool was prepared in English and then translated into the local Amharic language. Backward translation to English was also performed to check for consistency. Pretest was done on 5% of the total sample size and modifications were made accordingly. The final instrument was composed of socio-demographic characteristics, media exposure, health belief model constructs (perceived susceptibility, perceived severity, perceived benefit, perceived barrier), knowledge about COVID-19, preventive behaviors toward COVID-19, and willingness to pay for COVID-19 vaccine sections.

Data collection and supervision were done by public health professionals after they had received a 1-day training on the purpose of the study, procedures of data collection, and ethical considerations during data collection. Safety precautions toward COVID-19 preventions were taken during the data collection process. Each returned instrument was reviewed for completeness and consistency on daily basis.

### Measurements

#### Willingness to pay

It was the outcome variable of the study and defined as the maximum amount of money that teachers were willing to pay for the COVID-19 vaccination. To evaluate willingness to accept (WTA) and willingness to pay (WTP) for COVID-19 vaccination, it was hypothesized that a COVID-19 vaccine had been developed and tested in human and showed a 95% efficacy to prevent COVID-19 in the non-immune population with a 5% chance to produce a mild adverse effect such as mild skin pain, skin rash, and fever [8]. A double bounded dichotomous contingent valuation method (CVM) was used [19]. The respondents were first asked whether they would be willing to pay for the COVID-19 vaccine (WTA). If they said “yes,” they were asked whether they were willing to first bid 200 Ethiopian Birr (ETB) (equivalent with USD 5.15 using a November 2020 exchange rate of USD1 = 38.5 ETB) and then, the bid was either increased by 50 ETB where the highest bid was 300 ETB or decreased by 50 ETP to the lowest bid (100 ETB). A participant who refused to pay at the lowest bid (100 ETB/US$ 2.6) was considered not willing to pay [8, 14].

#### Perceived susceptibility

Perceived susceptibility was defined as a person’s subjective perception of the risk of acquiring COVID-19 and it was measured by three five-point Likert scale items. It was categorized into high and low based on the cutoff point computed using the demarcation threshold formula: {(highest score − lowest score)/2} + lowest score= [(15−3)/2] +3 = 9. Accordingly, participants who scored 9 and above were considered as having high perceived susceptibility to COVID-19 pandemic and those scored below 9 as having low perceived susceptibility to COVID-19 pandemic, (α = 0.78) [17, 18].

#### Perceived severity

Perceived severity refers to a person’s perception of the seriousness of contracting COVID-19 and is measured by three five-point Likert scale items. It was categorized into high and low based on the cutoff point computed using the demarcation threshold formula: {(highest score − lowest score)/2} + lowest score = [(15−3)/2] +3 = 9. Accordingly, participants who scored 9 and above were considered to have high perceived severity of COVID-19 pandemic, and those who scored below 9 were considered to have low perceived severity of COVID-19 pandemic, (α = 0.79) [17, 18].

#### Perceived benefit

Perceived benefit refers to a person’s perception of the effectiveness of the COVID-19 vaccine to prevent COVID-19 and is measured by three five-point Likert scale items. It was categorized into high and low based on the cutoff point computed using the demarcation threshold formula: {(highest score − lowest score)/2} + lowest score = [(15−3)/2] +3 = 9. Accordingly, participants who scored 9 and above were considered to have a high perceived benefit of taking the COVID-19 vaccine, and those who scored below 9 were considered to have a low perceived benefit from taking the COVID-19 vaccine, (α = 0.87) [13, 17, 18].

#### Perceived barriers

Perceived barriers refers to a person’s perception of the obstacles to receiving the COVID-19 vaccine and is measured by five items with a five-point Likert scale. It was categorized into high and low based on the cutoff point computed using the demarcation threshold formula: {(highest score − lowest score)/2} + lowest score = [(25−5)/2] +5 = 15. Accordingly, participants who scored 15 and above were considered to have a high perceived barrier to take COVID-19 preventive measures, whereas respondents who scored below 15 were considered to have a low perceived barrier to take COVID-19 preventive measures, (α = 0.73) [13, 17, 18].

#### Knowledge about COVID-19

Knowledge about COVID-19 refers to participant’s cognition about COVID-19 and it was measured by 11 items with three response categories (1 = True, 2 = False and 3 = I don’t know). A correct answer was coded as “1” point whereas, the incorrect and I don’t know responses were coded to “0.” The sum score was categorized based on the bloom’s cut-off as poor if the score was below 60% (< 9 points), moderate if the score was between 60 and 79% (9-10.9 points), and good if the score was 80% or above (≥ 10 points) (α = 0.87) [16].

#### COVID-19 preventive practice

COVID-19 preventive practice refers to the handwashing, physical distancing, and facemask-wearing practices and is measured by five items with a five-point Likert scale (1 = Never, 2 = Rarely, 3 = Sometimes, 4 = Most of the time, and 5 = All the time). The sum score was categorized based on the bloom’s cut-off as poor if practice score was < 60% (< 12 points), moderate if practice score was between 60 and 79% (12-13.9 points), and good if practice score was ≥ 80% (≥ 14 points) (α = 0.78) [15].

### Data processing and analysis

Data were coded and cleaned for completeness and consistency. Then, entered into EpiData version 4.6 and exported it into STATA version 14 statistical software for analysis. Descriptive analysis like medians, means, proportions, standard deviations, interquartile range, and frequencies were computed. Multicollinearity was checked using variance inflation factors (VIF) and the value of all variables was less than 5. Simple binary logistic regression analysis was employed and all independent variables with a p value less than 0.25 were entered in multiple binary logistic regression analysis. The overall model fitness of the final model was assessed with the Hosmer and Lemeshow goodness of fit test by which the mode showed adequate model fitness. A P value less than 0.05 and a 95% CI were used for declaring statistical significance.

### Ethical considerations

Ethical clearance was obtained from the Institutional Review Board of the University of Gondar a Ref. No: V/P/RCS/05/589/2020. Letter of the permission was obtained from the Gondar City administrative education office. After the purpose and objective of the study had been explained, written consent was obtained from each study participant. Any identifiers of the study participants were not recorded. Furthermore, the study was conducted following the Helsinki declaration.

## Result

A total of 301 school teachers participated in this study with a response rate of 99.6%. The mean age of participants was 39.5 (SD ± 8.7 years) with the age range of 21 to 64 years. More than half (59.1%) of the participants were males. The majority (63.5%) of them were public schools. Moreover, the majority (85.1%) of the participants perceived they were healthy and nearly one-fifth (18.9%) of the participants reported that they had been diagnosed with chronic disease (Table 1).

**Table 1:** Sociodemographic characteristics of school teachers in Gondar City, Northwest, Ethiopia (n = 301)

Variables	Frequency	Proportion
Age (years)		
Less than 40	53	17.61
40 and above	114	37.87
Sex		
Female	123	40.86
Male	178	59.14
Religion		
Orthodox Christian	251	83.39
Islam	31	10.3
Protestant	11	3.65
Catholic	4	1.33
Others	4	1.33
Highest educational level		
Diploma	60	19.93
BA/BSc degree	183	60.80
Master’s degree	58	19.27
Family size		
4 or below	163	54.15
Above 4	138	45.85
Monthly income		
< 5500	75	24.92
5500-6900	78	25.91
6901-7834	75	24.92
>7834	73	24.25
School type		
Government	191	63.46
Private	110	36.54
Listen to radio		
Less than once a week	160	53.16
At least once a week	141	46.84
Watch television		
At least once a week	13.95	86.05
Less than once a week	42	13.95
Read magazine/newspaper		
Less than once a week	238	79.07
At least once a week	63	20.93
Overall media exposure		
Yes	279	92.69
No	22	7.31
Presence of known chronic illness		
Yes	57	18.94
No	244	81.06
Self-rated health		
Healthy	256	85.05
Not Healthy	45	14.95
Know someone infected by COVID-19		
No	254	84.39
Yes	47	15.61

Note: *BA* Bachelor of Art, *BSC* Bachelor of Sciences

### Perceptions of COVID-19 and its vaccine

**Fig. 1:**
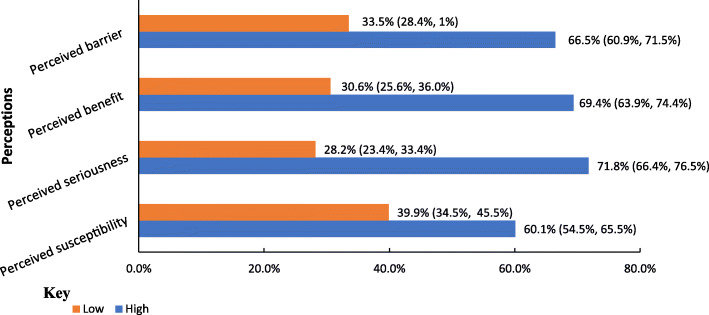
Perceptions of COVID-19 and its vaccine among school teachers in Gondar city, Northwest Ethiopia (n = 301)

The study participants’ perception about the COVID-19 and its vaccine was evaluated based on the health belief model constructs (perceived susceptibility to COVID-19, perceived seriousness of COVID-19, perceived barrier for COVID-19 vaccination, and perceived benefits of COVID-19 vaccination) (Fig. 1).

### Perceived susceptibility for COVID-19

**Fig. 2:**
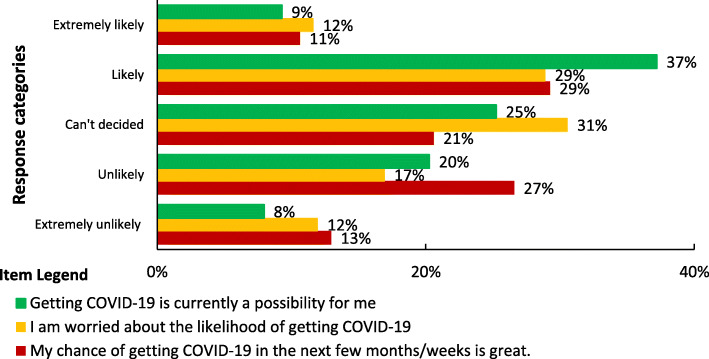
Perceived susceptibility for COVID-19 among school teachers in Gondar city, Northwest Ethiopia (n = 301)

More than half of (60.1%) the participants had high perceived susceptibility for COVID-19 (Fig. 1). About 45% (136), 41% (123), and 40% (121) of the participants believed getting COVID-19 is possible in their current status, perceived their chance of getting COVID-19 infection is high and worried about the likelihood of getting COVID-19 infection respectively (Fig. 2)

### Perceived seriousness of COVID-19

**Fig. 3:**
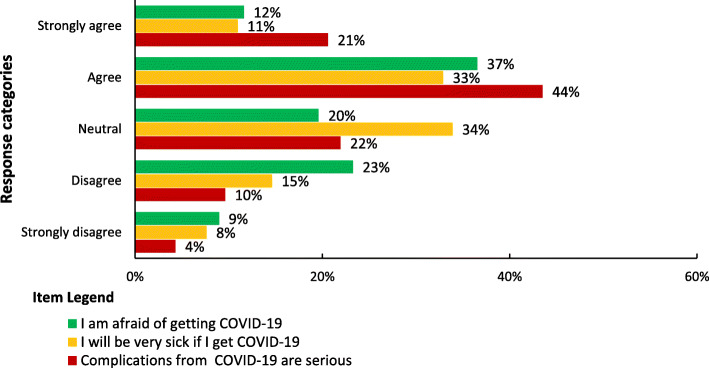
Perceived seriousness of COVID-19 among school teachers in Gondar city, Northwest Ethiopia (n = 301)

More than half (55%) of the participants perceived the complications from COVID-19 are serious. In addition to this, 44% and 49% of the participants perceived they will be very sick if they get COVID-19 and they were afraid of getting COVID-19 infection (Fig. 3). The majority 71.8% (216) of the participants had high perceived seriousness of the pandemic (Fig. 1).

### Perceived benefits of COVID-19 vaccine

**Fig. 4:**
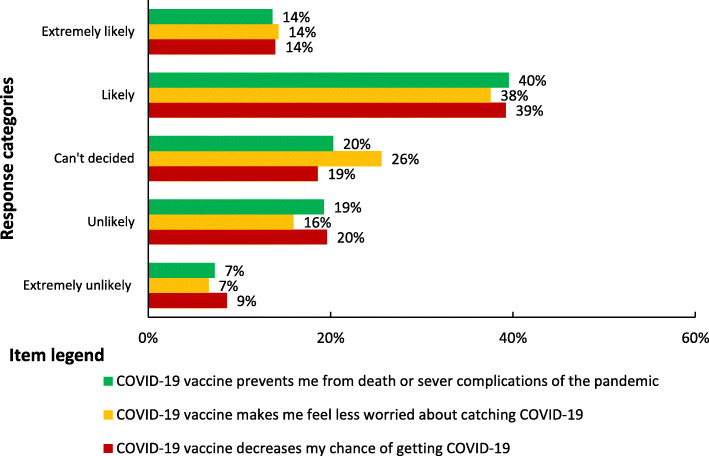
Perceived benefits of COVID-19 vaccination among school teachers in Gondar city, Northwest Ethiopia (n = 301)

The majority of the participants (69.4%) had higher perceived benefits of COVID-19 vaccination (Fig. 1). About 54% (163), 52% (157), and 53% (160) of the participants perceived COVID-19 vaccination will prevent them from death, decrease their chance of getting COVID-19 vaccination and let them feel less worried about the pandemic respectively (Fig. 4).

### Perceived barriers of COVID-19 vaccination

**Fig. 5:**
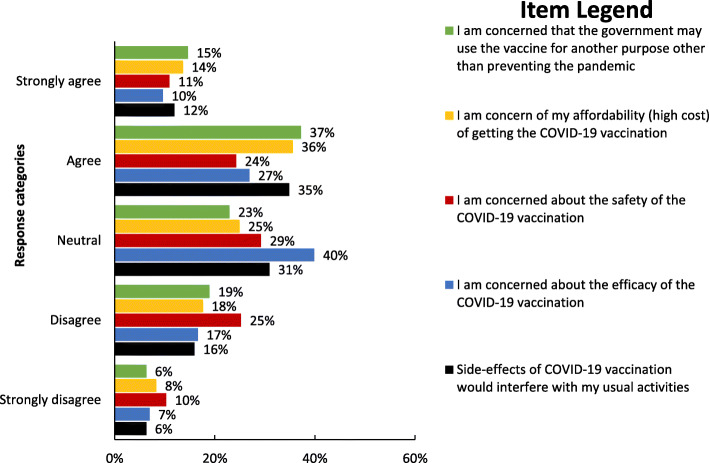
Perceived barriers of COVID-19 vaccination among school teachers in Gondar city, Northwest Ethiopia (n = 301)

From the total participants of the study, about 66.5% (200) of them had high perceived barriers to COVID-19 vaccinations (Fig. 1). Moreover, suspiciousness of the vaccine’s efficacy (67%), perceived cost of the vaccine (52%), and mistrusting the government that it may use the vaccine for another purpose (50%) were the commonest perceived barriers of COVID-19 vaccination mentioned by the respondents (Fig. 5).

### Knowledge and preventive practice toward COVID-19

Participants’ knowledge of COVID-19 was evaluated by computing each item score and graded based on the bloom’s cut-off into poor, moderate, and good knowledge. Given this, 38.2% (115), 28.6% (86), and 33.2% (100) of the participants had poor, moderate, and good knowledge about the COVID-19 pandemic respectively.

Moreover, school teachers’ COVID-19 preventive practice (social distancing, facemask wearing, and handwashing practice) was assessed. The overall COVID-19 preventive practice was computed and graded based on the bloom’s cut-off into poor, moderate, and good practice. In this regard, 45.9% (138), 30.9% (93), and 23.2% of the participants had poor, moderate, and good COVID-19 preventive practice respectively.

### Willingness to pay and acceptance for COVID-19 vaccine

About 40.8 (95% CI, 35.4, 46.5) of the participants were willing to accept the hypothetical vaccine against the COVID-19 pandemic. The mean and median willingness to pay for the COVID-19 vaccine were US$ 4.98 (± US$ 1.6) and US$ 5.2. From participants who were willing to accept the vaccine about one-fifth were willing to pay US$ 2.59. Moreover, the majority (88.3%) of the participant’s willingness to pay was less than or equal to US$ 6.49. From the total participants, less than half 36.9% (95% CI: (31.6%, 42.5%) of them were willing to pay for the vaccine if it would be available in the market with a price of 100 ETB (US$ 2.6). The most common reasons for not willing to pay were “I don’t trust the vaccine would be efficacious” (65%), “I can’t afford” (17.9%), and “I don’t worry about the pandemic” (12.6%) (Table 2).

**Table 2:** Willingness to accept and pay and reasons for not willing to pay for COVID-19 vaccine among school teachers in Gondar City, Northwest Ethiopia

	Proportion (%)	Std. err.	95% CI
Willingness to accept (n = 301)			
Yes	40.8	0.028	(35.4, 46.5)
No	59.2	0.028	(53.4, 64.5)
Willing to pay (n = 301)			
Yes	36.9	0.031	(31.6, 42.5)
No	63.1	0.031	(57.5, 68.4)
Willingness to pay in ETB (n = 111)	Frequency	Proportion (%)	
100	23	20.72	
150	12	10.81	
200	49	44.14	
250	14	12.61	
300	13	11.71	
Reasons for not willing to pay (n = 178)			
I don’t trust the vaccine	124	65.0	
I can’t afford the cost for the vaccine	34	17.89	
I don’t worry about the disease	24	12.63	
Other	8	4.21	

### Factors associated with willingness to pay for COVID-19 vaccine

In the first step, each independent variable was fitted to the outcome variable in the bivariable logistic regression model. Those variables showed a p value of less than 0.2 were entered into the final multivariable logistic regression model to identify factors associated with willingness to pay for the COVID-19 vaccine. As it is depicted in Table 3 below, male sex, being a private school teacher, high perceived susceptibility to COVID-19, high perceived seriousness of COVID-19, and high perceived benefit of COVID-19 vaccine were statistically significant factors associated with WTP for COVID-19 vaccine.

**Table 3:** Factors associated with willingness to pay for COVID-19 vaccine among school teachers in Gondar City, Northwest, Ethiopia (n = 301)

Independent variables	Willingness to pay		COR (95% CI)	Sig.	AOR (95% CI)
	No	Yes			
Sex					
Male	103 (57.9)	75 (42.1)	1.76 (1.10, 2.87)	0.024	3.23 (1.70, 6.14) **
Female	87 (70.7)	36 (29.3)	1		1
Age					
< 40	95 (63.3)	55 (36.7)	1		
≥ 40	95 (62.9)	56 (37.1)	1.02 (0.64, 1.62)	0.94	
Educational status					
Diploma	32 (53.3)	28(46.7)	1		1
BSc/BA degree	121 (66.1)	62 (33.9)	0.59 (0.32, 1.06)	0.08	0.68 (0.31, 1.51)
Master’s degree	37 (63.8)	21 (36.2)	0.65 (0.31, 1.35)	0.25	0.64 (0.21, 1.95)
Religion					
Christian	165 (62.0)	101 (38.0)	1		
Islam	21 (67.7)	10 (32.3)	0.78 (0.35, 1.71)	0.54	
School type					
Private	51 (46.4)	59 (53.6)	3.09 (1.89, 5.06)	0.00	3.27 (1.76, 6.10)**
Government	139 (72.8)	52 (27.2)	1		1
Media exposure					
No	17 (77.3)	5 (22.7)	1		1
Yes	173 (62.0)	106 (38.0)	2.08 (0.75, 5.81)	0.16	2.93 (0.89, 9.63)
Family size					
4 or below	108 (66.3)	55 (33.7)			
> 4	82 (59.4)	56 (40.6)	1.34 (0.84, 2.14)	0.22	
Monthly income (ETB)					
< 5500	38 (50.7)	37 (49.3)	1.48 (0.77, 2.83)	0.24	1.42 (0.54, 3.70)
5500-6900	49 (62.8)	29 (37.2)	0.89 (0 .47, 1.73)	0.75	0.93 (0.37, 2.31)
6901-7834	59 (78.7)	16 (21.3)	0.41 (0.20, 0.84)	0.02	0.56 (0. 22, 1.43)
> 7834	44 (60.3)	29 (39.7)	1		1
Had known chronic illness					
Yes	34 (59.7)	23 (40.3)	1.20 (0.66, 2.16)	0.55	
No	156 (63.9)	88 (36.1)	1		
Knew someone infected with COVID					
Yes	25 (53.2)	22 (46.8)	1.63 (0.87, 3.10)	0.13	1.44 (0.68, 3.0)
No	165 (65.0)	89 (35.0)	1		1
Self-rated health					
Healthy	160 (62.5)	96 (37.5)	1.20 (0.61, 2.34)	0.59	
Not healthy	30 (66.7)	15 (33.3)	1		
Perceived susceptibility					
High	103 (56.9)	78 (43.1)	2.0 (1.21, 3.28)	0.006	2.69 (1.38, 5.24)*
Low	87 (72.5)	33 (27.5)	1		1
Perceived seriousness					
High	125 (57.9)	91 (42.1)	2.36 (1.34, 4.18)	0.003	4.04 (1.80, 9.1) **
Low	65 (76.5)	20 (23.5)	1		1
Perceived benefit					
High	115 (55.0)	94 (45.0)	3.60 (2.0, 6.52)	0.00	3.0 (1.41, 6.34) **
Low	75 (81.5)	17 (18.5)	1		1
Perceived barrier					
High	124 (62.0)	76 (38.0)	1.15 (0.70, 1.91)	0.57	
Low	66 (65.4)	35 (34.6)	1		
Knowledge of COVID-19					
Poor	77 (67.0)	38 (33.0)	1		
Moderate	50 (58.1)	36 (41.9)	1.46 (0.82, 2.60)	0.19	1.23 (0.59, 2.57)
Good	63 (63.0)	37 (37.0)	1.19 (0.68, 2.09)	0.54	0.94 (0.46, 1.90)
COVID-19 preventive practice					
Poor	85 (61.6)	53 (38.4)	1		
Moderate	64 (68.8)	29 (31.1)	0.72 (0.42, 1.30)	0.27	
Good	41 (58.6)	29 (41.4)	1.13 (0.63, 2.03)	0.67	

Note:

*Significant at p value < 0.05

**Significant at p value < 0.001

Male teachers were 3.23 times more likely willing to pay for the COVID-19 vaccine compared to females (AOR = 3.23, 95% CI, (1.70, 6.14)). Teachers from private schools were 3.27 times more likely willing to pay for the COVID-19 vaccine than teachers who were from public schools (AOR = 3.27, 95% CI, (1.76, 6.10)). Regarding perceptions of school teachers toward COVID-19 and its vaccine, teachers with high perceived susceptibility to COVID-19 (AOR = 2.69 95% CI, (1.38, 5.24)), high perceived seriousness of COVID-19 (AOR = 4.04 95% CI, (1.80, 9.1)) and high perceived benefit of COVID-19 vaccine(AOR = 3.0, 95% CI (1.41, 6.34) were more likely willing to pay for COVID-19 vaccine compared to teachers with low perceived susceptibility to COVID-19, low perceived serious of COVID-19, and low perceived benefit of COVID-19 vaccination respectively (Table 3).

## Discussion

The main aim of this study was to assess willingness to pay for COVID-19 vaccination based on their perception of the pandemic and its vaccine. In this study, 40.8% of participants responded they would be willing to accept vaccination against COVID-19 infection if it were proven effective and safe. This figure is below the global acceptance range (55-90%) [20], and other studies which were done in Australia [21], Greek [22], Chile [14], the USA [13], and Japan [10]. However, it is higher than the finding reported in France and comparable to findings of studies from Spain and Sweden [23]. In addition to this, the present study also revealed that only 36.9% of participants were willing to pay for the vaccine. This finding is very low when compared to previous studies done in Indonesia [8] and China [24], where 78.3% and 89.7% of their participants were willing to pay for the COVID-19 vaccine respectively. This discrepancy may be accounted for variations in the spread of the pandemic across the countries. For instance, COVID-19 cases reported in Indonesia are 86.8% higher cases that have been diagnosed in Ethiopia [25]. This can influence the participant’s perceptions of the importance of the vaccine and ultimately their willingness to pay for it. People pay for something when they look at the benefits they can get from it. Given this, participants of this study may have lower perceived benefit of the vaccine than the participants from Indonesia due to the higher COVID-19 infection spread in Indonesia compared to Ethiopia. In this study, the median willingness to pay was 6 times lower than the median willingness to pay reported from Indonesia [8] and Malaysia [26], 2.5 times lower than the median willingness to pay reported from China [24], and the mean WTP of this study is 47.4 times lower than the mean willingness to pay reported from Chile [14]. This can be explained by the fact that the per capita income of Ethiopia is lower than those countries where the aforementioned previous studies were conducted. For instance, the per capita income of Ethiopia is 94.4% lower than that of Chile [27]. Moreover, context-specific factors such as perception of the severity and risk of diseases, which varied across disease types or locations may be another reason for the observed [7, 24, 26]. For example, the perceived risk of getting sick by COVID-19 was 39.1% higher among Chile population compared to this study’s participants [7]. Therefore, this could be the reason why WTP of this study participants was lower than in Chile [27].

The present study revealed that the willingness to pay was affected by various factors including the sex of the participant, the type of school where participants were working for, and participant’s perceptions about the pandemic and its vaccine. Male participants were two times more likely willing to pay than females. This finding is inconsistent with previous studies done to assess WTP for the COVID-19 vaccine in different parts of the world. However, it is in line with a study done in Japan [10]. The reason for this gender variation may be because males are more likely to have additional income sources than females in the local context. Consequently, they may have a higher willingness to pay than females. Moreover, teachers from private schools were also more likely to pay for the vaccine. This may be due to private school teachers may be paid a higher salary [8] or in the local context, almost all students who are attending their classes in private schools are students from wealthier families. In this case, teachers working in such kinds of schools may be expected to be vaccinated against the pandemic for the safety of students regardless of their willingness.

This study also claimed that participants with a high perceived risk of COVID-19 infection were more likely willing to pay for vaccination against the pandemic. This finding was further supported by previous studies reported from Malaysia [26] and Indonesia [8]. Human beings are rational, i.e., if they think they are vulnerable, they will take measures to avoid their risk. This may be the reason why those participants with a high perceived risk of COVID-19 were more likely to be willing to pay for the vaccine. Furthermore, the perceived benefits of the COVID-19 vaccine emerged as another factor affecting participant’s WTP. This finding is in line with a previous survey from Japan, where the high perceived benefit of the vaccine was associated with willingness to accept the vaccine [10]. This is may be because individuals’ perception of the positive consequences (benefit) that are caused by taking an action (vaccination), enhances their readiness of taking the recommended action, WTP [28].

One of the limitations of this study is a bias associated with the use of hypothetical WTP before the actual availability of the vaccine. The use of WTP in real contexts would reflect consumers’ actual valuation of the vaccine. Lack of generalizability to the community should also be noted since the study was institution-based. Furthermore, though contingency valuation method (CVM) is widely used approach to assess WTP, some authors postulate that biases can be generated by the respondent’s lack of understanding of the contingent market [29]. Indeed, casual inferences may not be possible due to the cross-sectional nature of the study. Therefore, the findings of this survey should be interpreted in light of the abovementioned limitations.

Notwithstanding the above limitations, this study attempted to assess a contemporary issue that could open the door for successive researchers to conduct further community-based studies. This study can also provide relevant information to those working for the production and/or distribution of COVID-19 vaccination. Moreover, teachers are a special group of population that are expected to be a role model to their students and to their community at large. If the acceptance of the COVID-19 vaccine low among this population, it is easy to guess that the COVID-19 vaccine acceptance would be low in the general community, even though community-based studies are required to verify. Furthermore, the findings of this study suggested that the concerned bodies are urgently required to design programs that promote vaccine acceptance among school teachers. Otherwise, it would be very challenging to create the expected herd immunity in the community. Currently, it is estimated that perhaps 60% of individuals need to be immune to attain herd immunity for COVID-19 [30]. According to the present study, it is unlikely that herd immunity through vaccination could be obtained without financial subsidies to vaccination and vaccine promotion activities.

### Conclusion and recommendations

Willingness to accept and willingness to pay for COVID-19 was very low among school teachers. Sex, school type, perceived risk (susceptibility and seriousness) of COVID-19, and perceived benefit of COVID-19 vaccine were important factors associated with school teachers’ willingness to pay. The result indicated that the government needs to subsidize the cost of the vaccination whenever it becomes available in the market. Besides, health communication interventions targeted at modifying perceptions about COVID-19 and its vaccine are urgently required to improve COVID-19 vaccine acceptability and WTP. Indeed, community-based researches are required to assess the public’s acceptance and willingness to pay for COVID-19.

## Data Availability

The datasets used and/or analyzed during the current study are available from the corresponding author on reasonable request (Kegnie Shitu, E-mail: kegnsh@gmail.com).

## Abbreviations

AOR: Adjusted odds ratio
CI: Confidence interval
COR: Crude odds ratio
COVID-19: Coronavirus disease
WTA: Willingness to accept
WTP: Willingness to pay
