# Correction to: Acceptance and willingness to pay for COVID-19 vaccine among school teachers in Gondar City, Northwest Ethiopia

**DOI:** 10.1186/s41182-021-00354-8

**Published:** 2021-08-20

**Authors:** Kegnie Shitu, Maereg Wolde, Simegnew Handebo, Ayenew Kassie

**Affiliations:** Department of Health Education and Behavioral Sciences, Institute of Public Health, College of Medicine and Health Sciences, University of Gondar, Gondar, Ethiopia

## Correction to: Trop Med Health (2021) 49:63 10.1186/s41182-021-00337-9

Following publication of the original article [1], the authors identified errors in the first name of Maereg Wolde and Simegnew Handebo.

The incorrect author names are: Meareg Wolde and Simegniew Handebo.

The correct author names are: Maereg Wolde, Simegnew Handebo.

The author group has been updated above and the original article [1] has been corrected.
